# miR-552 promotes the proliferation and metastasis of cervical cancer cells through targeting MUC15 pathway

**DOI:** 10.7150/jca.56098

**Published:** 2021-08-24

**Authors:** Xinxin Zhang, Yi Zhang, Lei Dou

**Affiliations:** 1 Department of Discipline Inspection Commission, China Medical University, Shenyang 110001, Liaoning, China.; 2 Department of Gynecology, the First Affiliated Hospital of China Medical University, Shenyang 110001, Liaoning, China.

**Keywords:** cervical cancer, miR-552, MUC15, proliferation, metastasis

## Abstract

Accumulating evidence shows that microRNAs (miRNAs) play key roles in tumorigenesis, progression, recurrence and drug resistance of malignant tumors. The tumor-promoting role of miR-552 has been evidenced in multiple tumors. Yet, the relevance of miR-552 in cervical cancer remains undetermined. This study aimed to investigate the role of miR-552 in cervical cancer proliferation and metastasis. Herein, we for first found that miR-552 expression was upregulated in cervical cancer tissues compared with their normal controls. Functional assays revealed that miR-552 promoted the proliferation and metastasis of cervical cancer cells. Mechanically, bioinformatics and luciferase reporter analysis identified MUC15 as a direct target of miR-552. Reduced MUC15 expression was detected in cervical cancer, and MUC15 overexpression exhibited a tumor-suppressive effect. MUC15 restoration partially abolished the discrepancy of growth and metastasis capacity between miR-552 overexpression cervical cancer cells and control cells. Taken together, these data demonstrate that miR-552 acts as a potential oncogene miRNA in cervical cancer, which exerts its function through targeting MUC15.

## Introduction

Cervical cancer is the fourth most common malignances and third leading cause of cancer deaths in women globally 1. Persistent infection of human papillomavirus (HPV) is considered as an important cause for cervical cancer initiation 2. The prognosis of cervical cancer patients has significantly improved due to the advance in cancer diagnostic technology and medical treatment. However, the outcome of patients with regional and distant metastasis is still unsatisfactory 3, 4. Therefore, it's urgent to explore the underlying mechanism of cervical cancer and find the new therapeutic target to improving the clinical outcome of patients suffering from cervical cancer.

MicroRNAs (miRNAs) is a group of small non-coding RNA molecule which containing about 22 nucleotides. miRNAs downregulate protein expression by targeting molecules 3'-UTR 5, 6. Increasing evidence shows that miRNAs play important function in tumorigenesis, progression, recurrence and drug resistance of malignant tumors 7, 8. Numerous researchers found that targeting specific miRNAs effectively restrains the initiation and progression of tumors, findings that suggest miRNAs are potential therapeutic targets for tumors 9, 10. In human cervical cancer, numbers of miRNAs were found to be abnormal expressed during its initiation and progression, including miR-205, miR-210-3p, miR-216a-3p, etc. 11-13. miR-552 is a newly discovered miRNA, its function and mechanism of action in biological processes and diseases are not completely known. Previous studies showed that miR-552 promotes laryngocarcinoma cells proliferation and metastasis by targeting p53 pathway 14. Zhao etc. found that miR-552 promotes ovarian cancer progression by regulating PTEN pathway 15. Moreover, Han etc. also found that miR-552 regulates liver tumor-initiating cell expansion and sorafenib resistance via targeting PETN/AKT pathway 16. However, the role of miR-552 in cervical cancer was unclear.

In the present study, we for first found that miR-552 was upregulated in human cervical cancer tissues and cell lines. Biological function study demonstrated that miR-552 knockdown inhibited cervical cancer proliferation and metastasis. Conversely, forced miR-552 expression facilitated cervical cancer proliferation and metastasis. Further mechanism study revealed that MUC15 was a direct target of miR-552. Our data also showed that MUC15 was downregulated in cervical cancer tissues and cell lines. MUC15 overexpression suppressed cervical cancer proliferation and metastasis. In concluding, our results highlighted the importance of miR-552 in promoting the proliferation and metastasis of cervical cancer cells via MUC15 pathway.

## Materials and Methods

### Collection of clinical tissue specimens

Human cervical cancer tissues and adjacent noncancerous tissues were obtained from 30 cervical cancer patients at the First Affiliated Hospital of China Medical University (Shenyang, Liaoning, China). The inclusion criteria were as follows: (1) The patients were diagnosed with cervical cancer; (2) No previous anti-tumor treatment. The exclusion criteria were as follows: (1) Patients with heart, lung, brain, kidney dysfunction; (2) Patients with other malignancies. Detailed clinicopathological features of these patients are described in supplementary Table 1. Written informed consent about tissue donation for study purposes was obtained from all the participants before the surgery. The study design was reviewed and approved by the Clinical Research Ethics Committees of the First Affiliated Hospital of China Medical University, and all experimental methods were carried out in accordance with the guidelines of the Declaration of Helsinki.

### Cell lines and cell culture

Human cervical carcinoma cell lines, including Ca_Ski, C-33A, HeLa, and SiHa, were obtained from the American Type Culture Collection (Manassas, VA). The human normal cervical epithelial immortalized H8 cells and 293T cells were provided by BeNa Culture Collection (BNCC, Kunshan City, China). All cells were cultured in the medium recommended by the manufacturers and grown at 37 °C with an atmosphere of 5% CO2 and 95% humidity in a cell incubator.

HeLa and SiHa cells were dissociated with 0.5% trypsin and seeded into six-well plates. HeLa and SiHa cells were infected with miR-552 sponge virus or miR-552 mimic virus and their control virus. HeLa and SiHa cells were infected with MUC15 overexpression virus and control virus. Then the stable infectants were screening by using puromycin as before 17. miR-552 sponge virus and miR-552 mimic virus was purchase from Shanghai GenePharma (Shanghai, China). MUC15 overexpression virus was purchased from Obio Technology Co. (Shanghai, China).

### Cell proliferation assays

For CCK8 assay, HeLa miR-552 sponge/mimic or SiHa cells miR-552 sponge/mimic and their control cells were seeded in 96-well plates (3×10^3^ cells per well). ATP activity was measured using a Cell Counting Kit-8 at indicated time points. ATP activity was measured using a Cell Counting Kit-8 at indicated time points. The procedure was as follows: The cell suspension (100 μl/well) was inoculated in a 96-well plate, and the plate was pre-incubated in a humidified incubator at 37 °C for 1 hour. This was followed by the addition of 10 μl of the CCK-8 solution to each well of the plate, and incubation of the plate for 1 h in the incubator. Finally, the absorbance was measured at 450 nm using a microplate reader (Synergy H1; BioTek Instruments, Inc., Winooski, VT, USA) 18.

For colony formation assay, HeLa miR-552 sponge/mimic or SiHa cells miR-552 sponge/mimic and their control cells were cultured in 12-well plates (3×10^3^ cells/well). The cells were incubated at 37 °C for 7 days and then fixed with 10% neutral formalin for >4 h. The cells were dyed with crystal violet (Beyotime, Haimen, China). The cells were photographed under a microscope (Olympus, Tokyo, Japan).

### Cell cycle assays

HeLa miR-552 sponge or SiHa cells miR-552 sponge and their control cells were planted into 6-well plates for 48 hours. Using trypsin to digest cells firstly and then centrifugation. Wash cell pellet with ice-cold PBS twice. Centrifugate the cells 600g for 5 minutes and transfer the tube to ice. Slowly resuspend the cells with ice-cold 70% ethanol in distilled water. Place cells at -20 °C before staining and analysis. Centrifugate the cells 1000g for 5 minutes at 4 °C. Remove the ethanol and resuspend cells in 1 mL ice-cold PBS. Centrifugate the cells 500g for 10 minutes at 4 °C and remove PBS. Then rewashed with 1 mL ice-cold PBS for twice. Then the cells were stained with Propidium Iodide (PI) (40 mg/mL, Abbkine, Inc, China) and RNase A (250 mg/mL, Roche Diagnostics) for 30 minutes at 37 °C in dark. Data were collected using a Molflo XDP (Beckman Coulter, Inc.250 S.Kraemer Boulevard Brea, CA 92821, USA) equipped with a Spectraphysics argon ion laser and analyzed using Summit (Beckman Coulter, Inc.250 S.Kraemer Boulevard Brea, CA 92821, USA). Results represent a minimum of 20,000 cells assayed for each sample.

### Apoptosis Assay

HeLa miR-552 sponge or SiHa cells miR-552 sponge and their control cells were cultured in six-well plate for 48 hours, followed by staining with Annexin V and 7-AAD for 15 minutes at room temperature in the dark. Apoptotic cells were determined by an Annexin VFITC Apoptosis Detection Kit I (BD Pharmingen, San Diego, CA) and detected by flow cytometry according to the manufacturer's instructions.

### Wound healing assay

For wound healing assay, monolayers of cells were wounded by scraping with a plastic pipette tip and rinsed several times with medium to remove dislodged cells. Cells that had migrated into the wound area were photographed.

### Cell migration assays

For cell migration experiments, 2×10^5^ HeLa miR-552 sponge/mimic or SiHa cells miR-552 sponge/mimic and their control cells were seeded into the upper chamber of a polycarbonate transwell in serum-free DMEM medium. The lower chamber was added with DMEM medium containing 20% FBS as chemoattractant. The cells were incubating for 24 hours and the chamber was fixed with 10% neutral formalin for >4 hours. The cells were dyed with crystal violet (Beyotime). The cells were then counted under a microscope (Olympus) and the cell number is expressed as the average number of the cells in each field.

### Cell invasion assays

For cell invasion experiments, 2×10^5^ HeLa miR-552 sponge/mimic or SiHa cells miR-552 sponge/mimic were seeded into the upper chamber of a polycarbonate transwell in serum-free DMEM medium. The lower chamber was added with DMEM medium containing 20% FBS as chemoattractant. The cells were incubating for 36 hours and the chamber was fixed with 10% neutral formalin for >4 hours. The cells were dyed with crystal violet (Beyotime). The cells were then counted under a microscope (Olympus) and the cell number is expressed as the average number of the cells in each field.

### Luciferase reporter assays

The cDNA fragment of MUC15 3'-UTR that contained the wild-type or mutant miR-552 binding site was inserted into the miRNA reporter vector (Promega, Madison, WI). Briefly, cervical cancer cells were co-transfected with miR-552 sponge, miR-552 mimic or miR-control and pMIR-reporter luciferase vector containing a specific sequence of wild-type or mutant MUC15 fragment, using siRNA transfection (Invitrogen, NY, USA). Cells were collected and lysed for luciferase detection 48 h after transfection. The relative luciferase activity was normalized against to the Renilla luciferase activity 19.

### Real-time PCR

Total RNA from cells or tissues was extracted using the TRIzol reagent (Takara) according to the manufacturer's instructions. Reverse transcription reactions for miRNAs were performed with SYBR PrimeScriptTM miRNA RT-PCR Kit (TaKaRa Bio Group, Shiga, Japan). U6 RNA was used as the internal control. All samples were normalized to the internal controls, and fold changes were calculated via the relative quantification method (2^-∆∆CT^). The miR-552 primer sequences were forward: 5' CCGCACAGGTGACTGGTTAGA 3', reverse: 5' GTGCAGGGTCCGAGGT 3', U6 primer sequences were forward: 5' CTCGCTTCGGCAGCACA 3', reverse: 5' AACGCTTCACGAATTTGCGT 3'.

The total cells RNA was extracted by using Trizol reagent (Invitrogen, 15596-018). Total cDNAs were synthesized by ThermoScript TM RT-PCR system (Invitrogen, 11146-057). The total mRNA amount presented in the cells was measured by RT-PCR using the ABI PRISM 7300 sequence detector (Applied Biosystems). The MUC15 primer sequences were forward: 5' AACCCATCTGTTTTCTCTAA 3', reverse: 5' AGATTGTATTATGCCCATTT 3'. The β-actin was used as reference for relative expression calculation and its primer sequences were forward: 5' GGCCCAGAATGCAGTTCGCCTT 3', reverse: 5' AATGGCACCCTGCTCACGCA 3'.

### Western Blotting assays

The western blotting analysis was used to detect protein MUC15, PARP and GAPDH expression. The proteins were collected as previous described 20. RIPA cell lysate containing protease inhibitor was used to lyse the cells for 30 min. After centrifugation at 12,000 g for 15min at 4 °C, the supernatant was obtained. After electrophoresis, the protein sample was transferred to a PVDF membrane, and the 5% skim milk powder was sealed at room temperature for 1 h. Rabbit antihuman MUC15 antibody (Abcam, Cambridge, MA), PARP antibody (Proteintech, Chicago, USA) or mouse anti-human GAPDH antibody (Proteintech, Chicago, USA) were added separately and incubated overnight at 4 °C. After washing with TBST, membranes were incubated with secondary antibody at room temperature for 2 h. TBST was used to clean the membrane 3 times. Bands were detected using the ECL Kit and b-Actin was used as a loading control.

### Statistical analysis

All statistical analyses were performed using GraphPad Prism (GraphPad Software, Inc. La Jolla, USA). Statistical analysis was carried out using t test or Bonferroni Multiple Comparisons Test: *p<0.05. A p value of less than 0.05 was considered statistically significant.

## Results

### High expression of miR-552 was observed in cervical cancer tissues and cells

**Figure 1:**
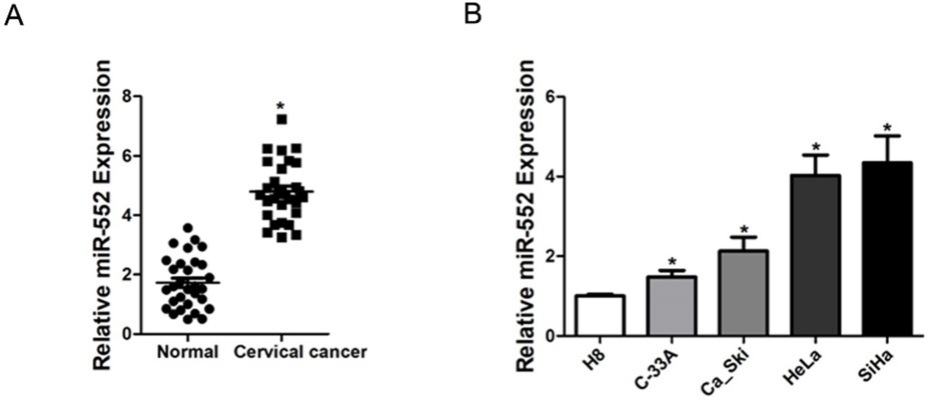
** Increased miR-552 expression in human cervical cancer. A.** Relative expression of miR-552 in human cervical cancer and normal tissues samples was determined by real-time PCR analysis (n=30) (*p < 0.05). **B.** The expression of miR-552 in cervical cancer cell lines (Ca_Ski, C-33A, HeLa, and SiHa) and normal H8 cervical epithelial cells was investigated via real-time PCR analysis (n=3, *p < 0.05).

To elucidate the role of miR-552 in the progression of cervical cancer, we first determined its expression pattern in tumor tissues of cervical cancer by real-time PCR. Compared with normal tissues, significant higher expression of miR-552 was detected in cervical cancer tissues (Figure 1A). Moreover, cervical cancer cell lines *in vitro* also showed increased miR-552 expression compared with normal H8 cervical epithelial cells (Figure 1B). Overall, these results suggest that miR-552 is an upregulated miRNA in cervical cancer.

### miR-552 promoted cervical cancer cells proliferation

**Figure 2:**
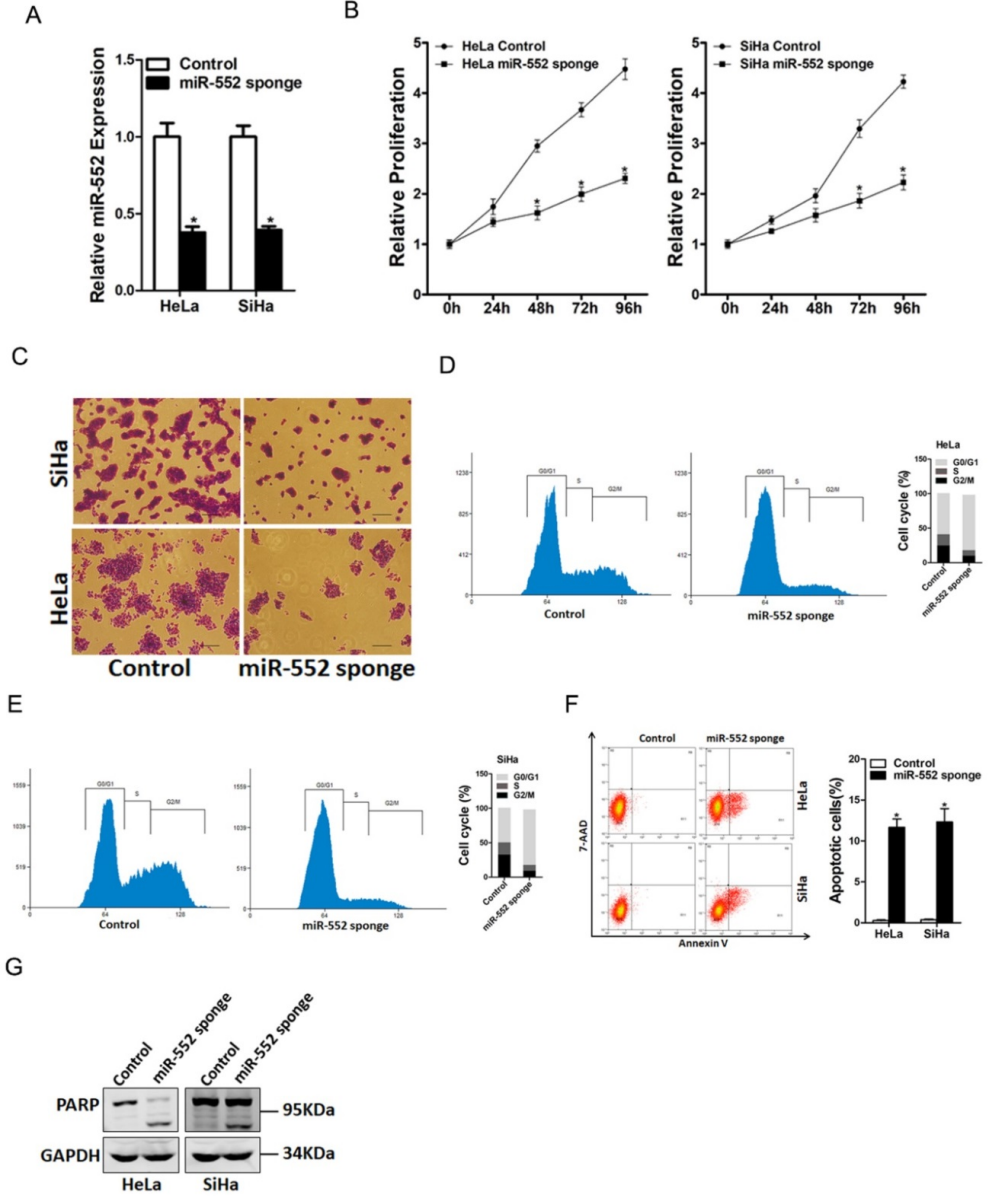
** miR-552 knockdown suppresses cervical cancer cells proliferation. A.** The interference effect of miR-552 in HeLa and SiHa cells was determined by real-time PCR analysis (n=3, *p < 0.05). **B.** Cell proliferation in HeLa miR-552 sponge or SiHa miR-552 sponge and their control cells was measured by using CCK-8 assays (n=6, *p < 0.05). **C.** Colony formation assays of HeLa miR-552 sponge or SiHa miR-552 sponge and their control cells (n=4, *p < 0.05). **D.** Cell cycle in HeLa miR-552 sponge and its control cells was assessed by flow cytometry (n=3, *p < 0.05). E. Cell cycle in SiHa miR-552 sponge and its control cells was assessed by flow cytometry (n=3, *p < 0.05). **F.** The apoptotic cells of miR-552 sponge and their control cells were assessed by flow cytometry (n=3, *p < 0.05). **G.** The cleave-PAPR expression in HeLa miR-552 sponge or SiHa miR-552 sponge and their control cells was determined by western bolt analysis.

To explore the biological function of miR-552 in regulating the malignant biological behaviors of cervical cancer cells, miR-552 gain-of-function and loss-of-function experiments were performed in cervical cancer cells *in vitro*. HeLa and SiHa cells were infected with miR-552 sponge virus or miR-552 overexpression virus. The knockdown and overexpression effect were determined by real-time PCR assay (Figure 2A and supplementary Figure 1A). CCK8 assay was used to examine the cell growth; the result showed that miR-552 interference inhibited cell growth in cervical cancer cells and ectopic miR-552 expression promoted cervical cancer cells growth (Figure 2B and supplementary Figure 1B). Next, the colony formation assay used to measure cell proliferation, the data showed that miR-552 knockdown cervical cancer cells formed less and smaller colonies (Figure 2C). Conversely, miR-552 overexpression cervical cancer cells formed much more colonies compared with control cells (supplementary Figure 1C). In addition, flow cytometry data showed a decreased S transition and a marked G0/G1 arrest in miR-552 interference cervical cancer cells (Figure 2D&E). Moreover, flow cytometry analysis also showed that miR-552 knockdown induced cervical cancer cells apoptosis (Figure 2F&G). Taken together, the above results showed that miR-552 promoted cervical cancer cells proliferation.

### miR-552 facilitated cervical cancer cells metastasis

**Figure 3:**
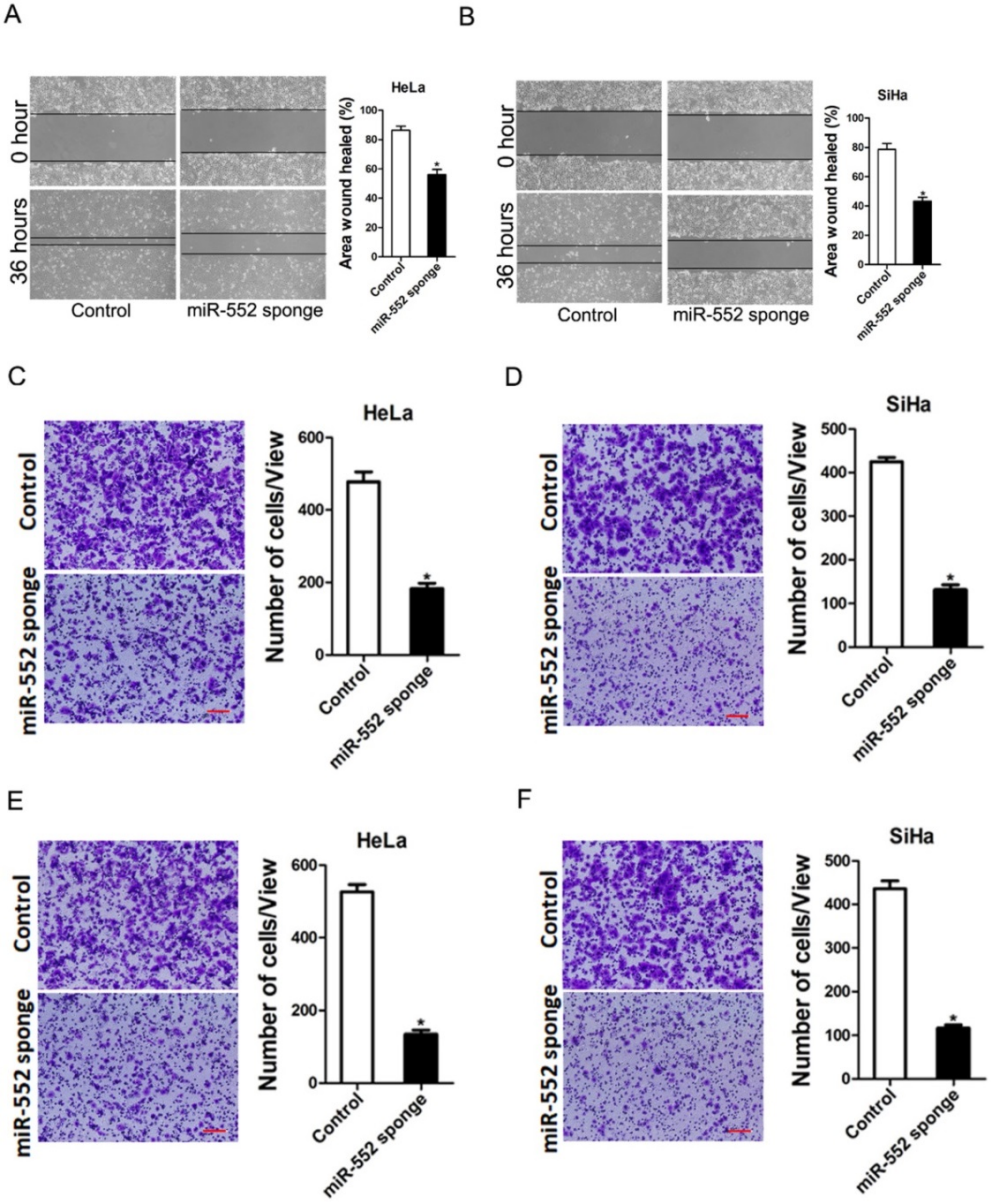
** miR-552 depletion inhibits cervical cancer cells migration and invasion. A.** Wound healing assay was performed to compare the migratory properties of HeLa miR-552 sponge and its control cells. Magnification, 100X; *p <0.05 (n=3). **B.** Wound healing assay was performed to compare the migratory properties of SiHa miR-552 sponge and its control cells. Magnification, 100X; *p <0.05 (n=3). **C.** The migration ability of HeLa miR-552 sponge and its control cells were performed utilizing polycarbonate membrane inserts in a 24-well plate. Scale bar: 20 µm (n=4, *p < 0.05). **D.** The migration ability of SiHa miR-552 sponge and its control cells were performed utilizing polycarbonate membrane inserts in a 24-well plate. Scale bar: 20 µm (n=4, *p < 0.05). **E.** The invasive capacity of HeLa miR-552 sponge and its control cells were analyzed using Matrigel-coated Boyden chamber. Scale bar: 20 µm (n=4, *p < 0.05). **F.** The invasive ability of SiHa miR-552 sponge and its control cells was analyzed using Matrigel-coated Boyden chamber. Scale bar: 20 µm (n=4, *p < 0.05).

Next, we also explore whether miR-552 influenced cervical cancer cells metastasis. As expected, scratch wound healing assay and transwell assay showed that miR-552 interference impaired the migration ability of cervical cancer cells (Figure 3A-D). Consistently, matrigel invasion chamber assay revealed that the invasion ability was impaired in miR-552 knockdown cervical cancer cells (Figure 3E&F). Moreover, miR-552 overexpression enhanced the migration and invasion ability of cervical cancer cells (supplementary Figure 2A&B). Collectively, our data demonstrated that miR-552 facilitated cervical cancer cells metastasis.

### miR-552 directly targeted MUC15 in cervical cancer cells

**Figure 4:**
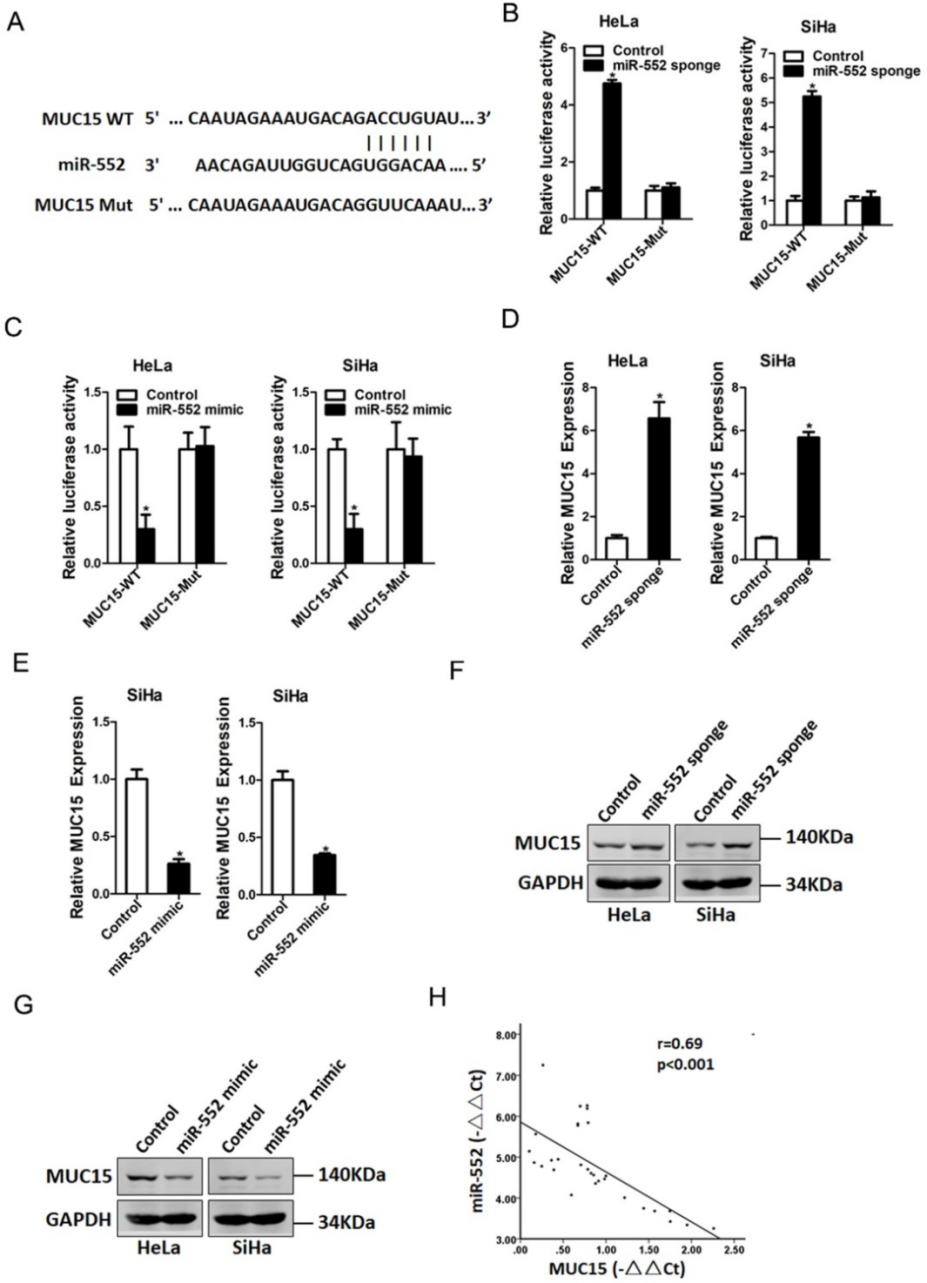
** MUC15 was a direct target of miR-552 in cervical cancer cells. A.** A potential target site for miR-552 in the 3'-UTR of human MUC15 mRNA, as predicted by the program Targetscan and miRBase. To disrupt the interaction between miR-552 and MUC15 mRNA, the target site was mutated. **B.** Luciferase reporter assays performed in HeLa miR-552 sponge or SiHa miR-552 sponge and their control cells transfected with wild-type or mutant MUC15 3'-UTR constructs (n=3, *p < 0.05). **C.** Luciferase reporter assays performed in HeLa miR-552 mimic or SiHa miR-552 mimic and their control cells transfected with wild-type or mutant MUC15 3'-UTR constructs (n=3, *p < 0.05). **D.** The mRNA expression of MUC15 was checked in HeLa miR-552 sponge or SiHa miR-552 sponge and their control cells by real-time PCR (n=3, *p < 0.05). **E.** The mRNA expression of MUC15 was checked in HeLa miR-552 mimic or SiHa miR-552 mimic and their control cells by real-time PCR (n=3, *p < 0.05). **F.** The protein expression of MUC15 was checked in HeLa miR-552 sponge or SiHa miR-552 sponge and their control cells by western blot. **G.** The protein expression of MUC15 was checked in HeLa miR-552 mimic or SiHa miR-552 mimic and their control cells by western blot. **H.** Significant correlation was observed between miR-552 and MUC15 expression in human cervical cancer tissues (n=30).

Next, we attempted to identify the potential target genes of miR-552 in cervical cancer cells. Bioinformatics analysis found that miR-552 has a putative binding site in MUC15 mRNA 3'-UTR (Figure 4A). To further explore whether miR-552 directly regulates MUC15 expression via interaction with its 3'-UTR, the wild-type or mutant MUC15 3'-UTR reporter plasmids were transfected into miR-552 sponge or miR-552 mimic cells and their control cervical cancer cells. It was found that luciferase activity was upregulated by interference of miR-552 in reporter gene construction containing wild-type 3'-UTR, but not in construction containing mutant 3'UTR (Figure 4B). Conversely, the luciferase activity was downregulated by overexpression of miR-552 in reporter gene construction containing wild-type 3'UTR, but not in construction containing mutant 3'-UTR (Figure 4C). Moreover, MUC15 mRNA expression was increased in miR-552 knockdown cervical cancer cells and decreased in miR-552 overexpression cervical cancer cells (Figure 4D&E). Consistently, MUC15 protein expression was also upregulated in miR-552 knockdown cervical cancer cells and downregulated in miR-552 overexpression cervical cancer cells (Figure 4F&G). There was a significant negative correlation between miR-552 and MUC15 mRNA expression in human cervical cancer tissues (Figure 4H).

### MUC15 inhibited cervical cancer cells proliferation and metastasis

**Figure 5:**
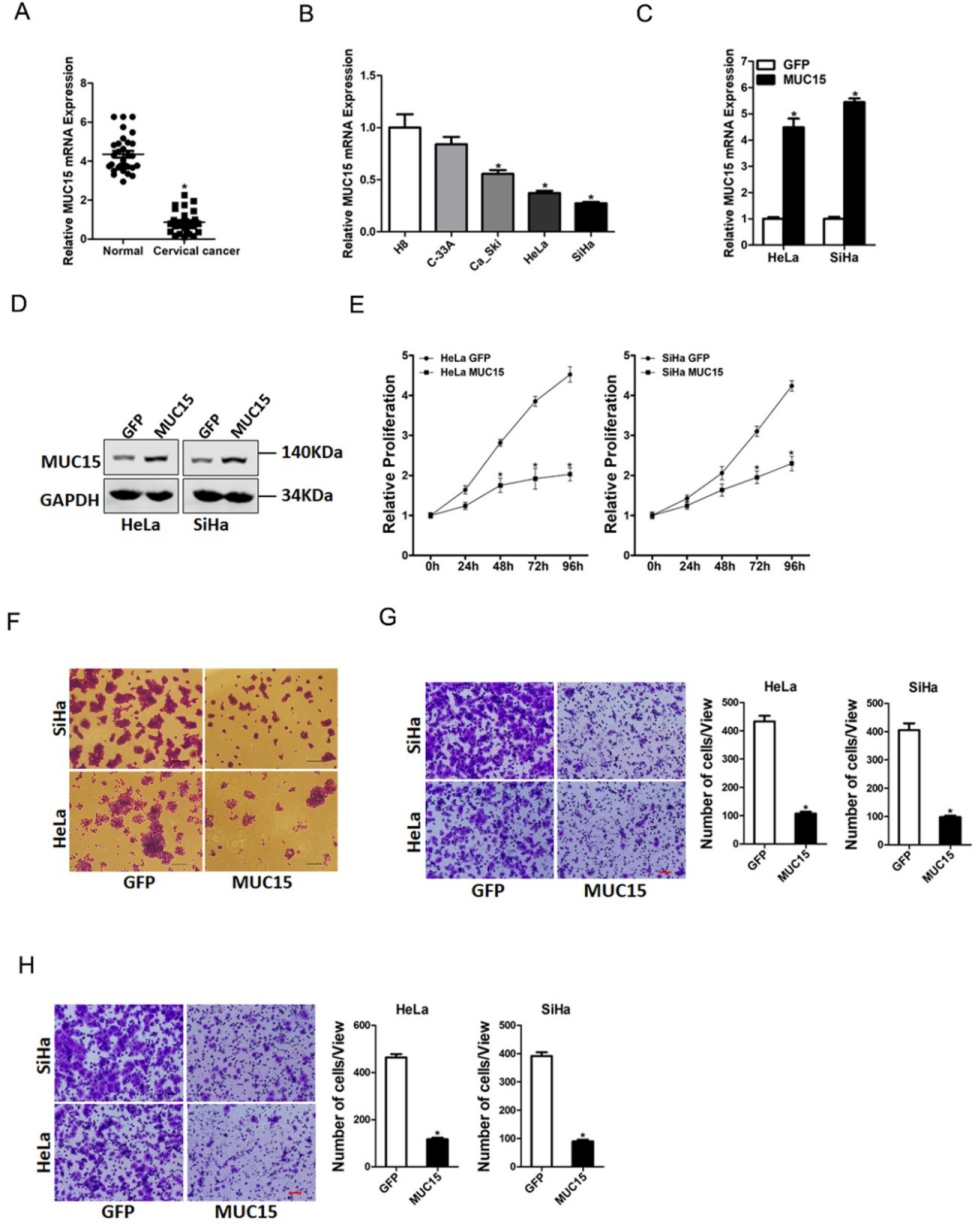
** MUC15 overexpression represses cervical cancer cells proliferation and metastasis. A.** Relative expression of MUC15 in human cervical cancer and normal tissues samples was determined by real-time PCR analysis (n=30) (p<0.05). **B.** The expression of MUC15 in cervical cancer cell lines (Ca_Ski, C-33A, HeLa, and SiHa) and normal H8 cervical epithelial cells was investigated via real-time PCR analysis (n=3, *p < 0.05). **C.** The overexpression effect of MUC15 in HeLa and SiHa cells was determined by real-time PCR analysis (n=3, *p < 0.05). **D.** The overexpression effect of MUC15 in HeLa and SiHa cells was determined by western blot analysis. **E.** Cell proliferation in HeLa MUC15 or SiHa MUC15 and their control cells was measured by using CCK-8 assays (n=6, *p < 0.05). **F.** Colony formation assays of HeLa MUC15 or SiHa MUC15 and their control cells (n=4, *p < 0.05). **G.** The migration ability of HeLa MUC15 or SiHa MUC15 and their control cells were performed utilizing polycarbonate membrane inserts in a 24-well plate. Scale bar: 20 µm (n=4, *p < 0.05). **H.** The invasive capacity of HeLa MUC15 or SiHa MUC15 and their control cells were analyzed using Matrigel-coated Boyden chamber. Scale bar: 20 µm (n=4, *p < 0.05).

To elucidate the potential role of MUC15 in the progression of cervical cancer, we first checked its expression pattern in tumor tissues of cervical cancer by real-time PCR. Compared with normal tissues, significant lower expression of MUC15 was detected in cervical cancer tissues (Figure 5A). Moreover, cervical cancer cell lines *in vitro* also showed decreased MUC15 expression compared with normal H8 cervical epithelial cells (Figure 5B). Next, HeLa and SiHa cells were infected with MUC15 overexpression virus. The overexpression effect was determined by real-time PCR and western blot assay (Figure 5C&D). CCK8 assay showed that MUC15 overexpression suppressed cell growth in cervical cancer cells (Figure 5E). The colony formation assay found that MUC15 overexpression cervical cancer cells formed less and smaller colonies (Figure 5F). Moreover, MUC15 overexpression impaired the migration and invasion ability of cervical cancer cells (Figure 5G&H). Collectively, our data demonstrated that MUC15 inhibited cervical cancer cells growth and metastasis.

### miR-552 promoted cervical cancer cells progression via targeting MUC15

**Figure 6:**
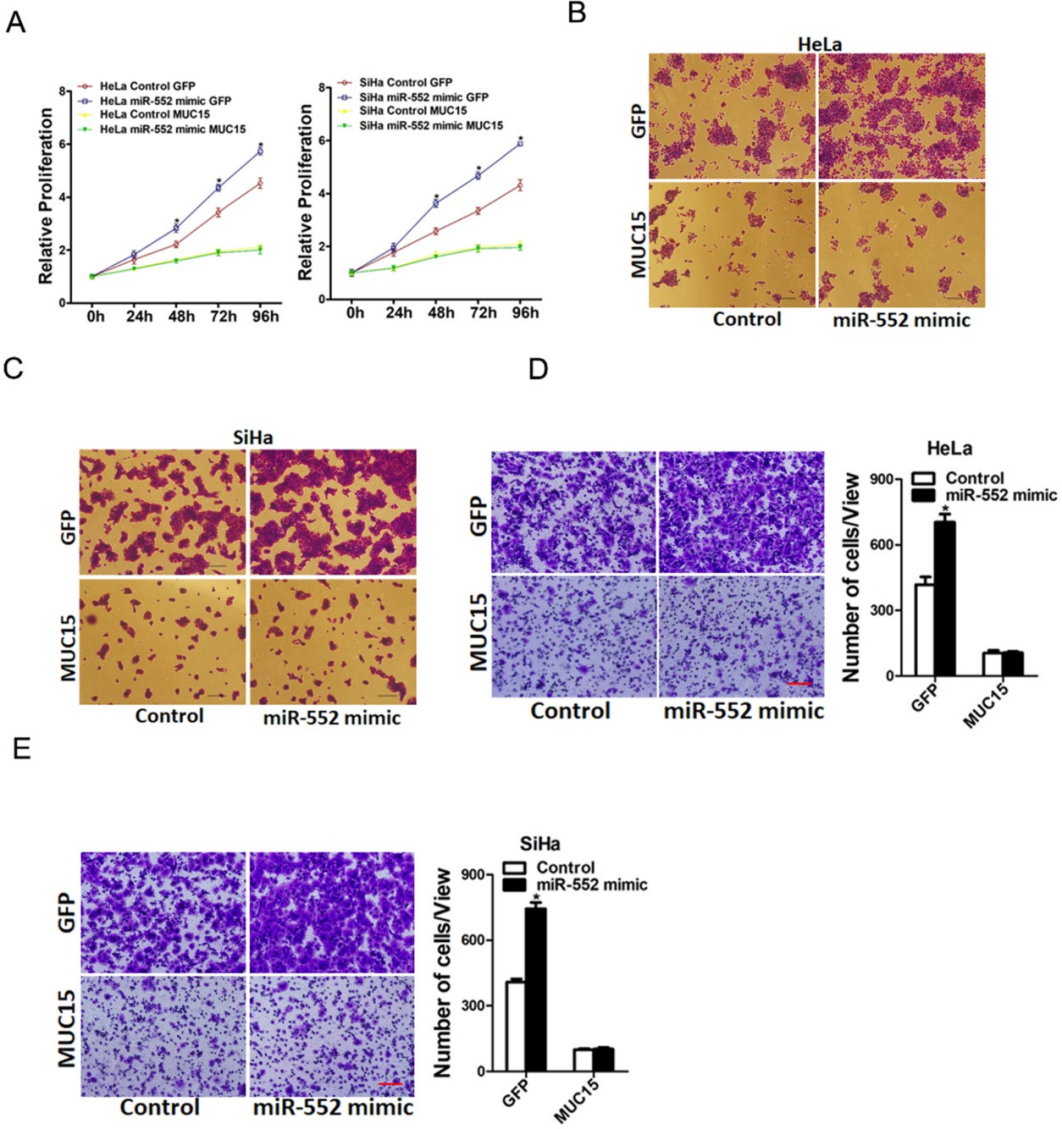
** miR-552 promotes cervical cancer cells progression via targeting MUC15. A.** HeLa miR-552 mimic or SiHa miR-552 mimic and their control cells were infected MUC15 overexpression virus and then subjected to CCK8 assay (n=6, *p < 0.05). **B.** HeLa miR-552 mimic and its control cells were infected MUC15 overexpression virus and then subjected to colony formation assay (n=4, *p < 0.05). **C.** SiHa miR-552 mimic and its control cells were infected MUC15 overexpression virus and then subjected to colony formation assay (n=4, *p < 0.05). **D.** HeLa miR-552 mimic and its control cells were infected MUC15 overexpression virus and then subjected to Invasion assay. Scale bar: 20 µm (n=4, *p < 0.05). **E.** SiHa miR-552 mimic and its control cells were infected MUC15 overexpression virus and then subjected to Invasion assay. Scale bar: 20 µm (n=4, *p < 0.05)

To further investigate the role of MUC15 in miR-552-mediated proliferation and metastasis of cervical cancer cells, miR-552 overexpression cervical cancer cells and control cells was infected with MUC15 overexpression virus. As expected, MUC15 overexpression diminished the distinct growth capacity between miR-552 overexpression cervical cancer cells and control cells (Figure 6A-C). Consistently, MUC15 overexpression also abrogated the discrepancy of metastasis between miR-552 overexpression cells and their control cells (Figure 6D&E). Taken together, the above results showed that MUC15 was involved in miR-552-mediated cervical cancer cells progression.

## Discussion

Accumulating evidence demonstrated that miRNAs participated in the regulation of cervical cancer proliferation, metastasis and recurrence. But there are still many unknown miRNAs in cervical cancer. Therefore, it's urgent to discover new functional miRNAs in cervical cancer. In the current study, we revealed a key role for miR-552 in cervical cancer. Our results confirmed miR-552 was an increased miRNA in cervical cancer and suggested a tumor-promoting role of miR-552 in cervical cancer. The oncogene effect of miR-552 on cervical cancer cell proliferation and invasion was associated with its regulatory effect on its target gene MUC15.

MiR-552 is a non-coding RNA consisting of 22 bases. Researchers have reported that miR-552 is abnormally expressed in colorectal cancer, osteosarcoma, ovarian cancer and hepatocellular carcinoma 21-23. In colorectal cancer, researchers found that increased expression of miR-552 acts as a potential predictor biomarker for poor prognosis of colorectal cancer 24. In liver cancer, miR-552 promotes the proliferation, migration and EMT of hepatocellular carcinoma cells by inhibiting AJAP1 expression. Moreover, miR-552 is also upregulated in liver tumor-initiating cells and promotes liver tumor-initiating cells expansion via PENT/AKT pathway. Overall, these findings support the notion that miR-552 is a tumor-promoting miRNA. However, whether miR-552 is involved in cervical cancer remains unknown. This study demonstrated that miR-552 is abnormally high-expressed in cervical cancer. Cell experiments confirmed that miR-552 can effectively promote the proliferation and metastasis of cervical cancer cells. MiR-552 can downregulate MUC15 protein expression by directly targeting MUC15 3'-UTR.

MUC15 belongs to one of several high-molecular-weight glycoprotein families that are often heavily O-glycosylated in either the cytoplasm or the membrane 25. Recent studies demonstrated a potential role for MUC15 in the pathogenesis and metastasis of cancers. For instance, MUC15 was observed highly expressed in colorectal adenocarcinomas and the oncogenic potential of human colon cancer cells 26. Conversely, MUC15 was downregulated in liver cancer and inhibited liver cancer metastasis by targeting EGFR/PI3K-AKT pathway 27. Currently, the potential role of MUC15 in cervical cancer remains largely unknown. In this study, decreased MUC15 expression was detected in cervical cancer. Functional assays revealed that MUC15 inhibited the proliferation and metastasis of cervical cancer cells. We also found that MUC15 was a direct target of miR-552 in cervical cells. miR-552 knockdown increased MUC15 mRNA and protein expression in cervical cancer cells. Conversely, miR-552 overexpression reduced MUC15 mRNA and protein expression in cervical cancer cells. miR-552 expression was negatively associated with MUC15 expression in cervical cancer tissues. More importantly, MUC15 overexpression could abrogate the distinct growth capacity or metastasis ability between miR-552 overexpression cervical cancer cells and control cells. Herein, we for first revealed that miR-552 promote cervical cancer proliferation and metastasis via directly regulating MUC15. These findings of the present study not only shed a new light on the mechanism of cervical cancer but suggest a potential therapeutic target against cervical cancer patients.

## Supplementary Material

Supplementary figures and tables.
