# Supplementary material for: miR-552 promotes the proliferation and metastasis of cervical cancer cells through targeting MUC15 pathway
Source: J Cancer. 2021 Aug 24;12(20):6094–104. doi: 10.7150/jca.56098 (PMC8425207; doi:10.7150/jca.56098)
Supplement: Supplementary file 1 — Supplementary figures and tables. [file jcav12p6094s1.pdf]

miR-552 promotes the proliferation and metastasis of cervical cancer cells through targeting MUC15 Pathway

Xinxin Zhang[1], Yi Zhang[1], Lei Dou[1,*]

[1]Department of Discipline Inspection Commission, China Medical University, Shenyang 110001, Liaoning, China.

[2]Department of Gynecology, the First Affiliated Hospital of China Medical University, Shenyang 110001, Liaoning, China.

Corresponding author: Dr. Lei Dou, Department of Gynecology, The First Affiliated Hospital of China Medical University, Shenyang 110001, China. Email address: doulei840416@163.com.

Running title: miR-552 drives cervical cancer cells progression.

Supplementary Figure 1

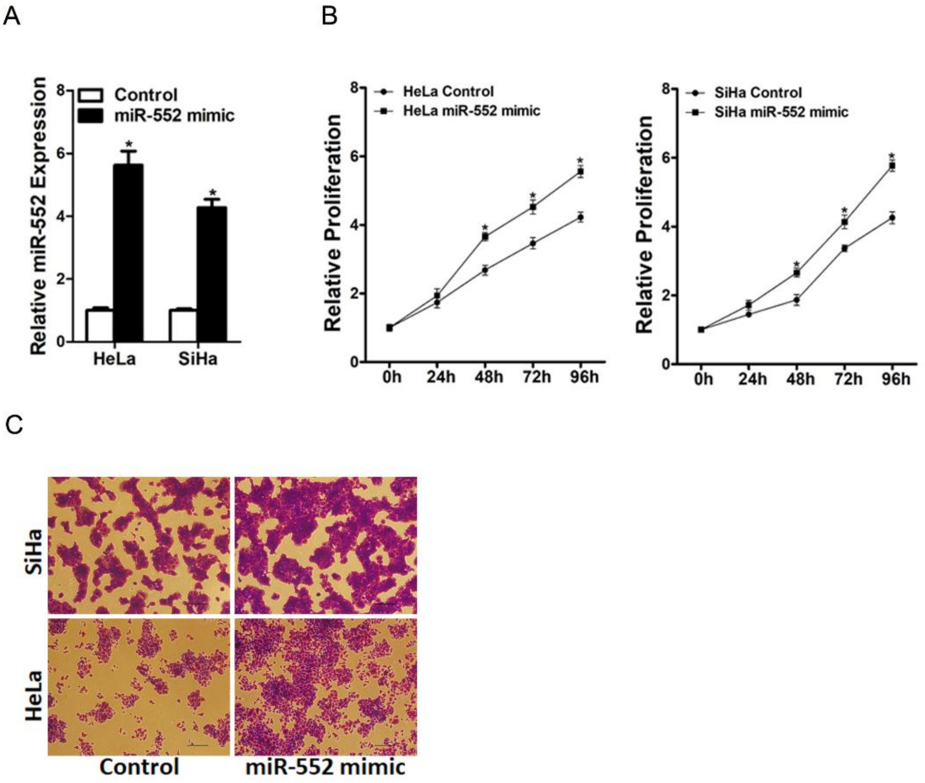

Supplementary Figure 1

A. The overexpression effect of miR-552 in HeLa and SiHa cells was checked by real-time PCR assay. (n=3, *$p < 0.05$)

B. Cell proliferation was measured using CCK-8 assays in HeLa miR-552 mimic or SiHa miR-552 mimic and their control cells. (n=6, *$p < 0.05$)

C. Colony formation assays of HeLa miR-552 mimic or SiHa miR-552 mimic and their control cells. (n=4, *$p < 0.05$)

Supplementary Figure 2

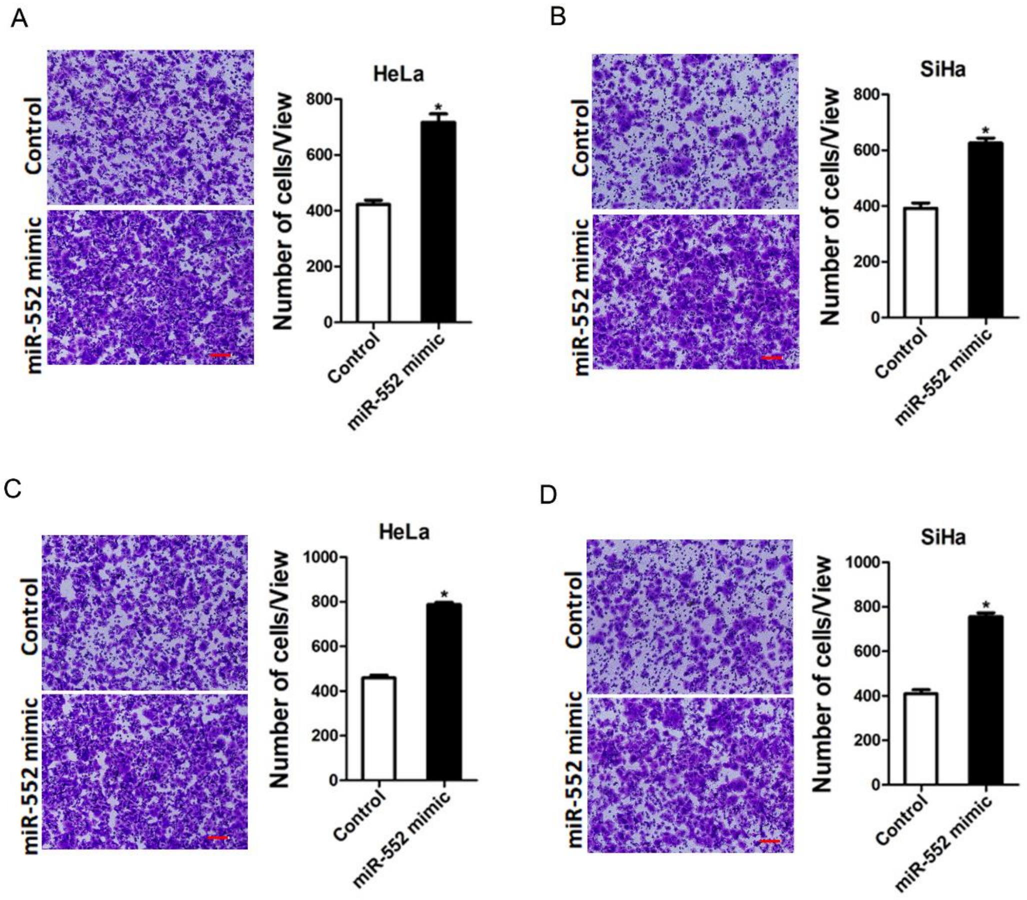

Supplementary Figure 2

A. The migration ability of HeLa miR-552 mimic and its control cells was performed utilizing polycarbonate membrane inserts in a 24-well plate. Scale bar=20 μm (n=4, *p < 0.05).

B. The migration ability of SiHa miR-552 mimic and its control cells was performed utilizing polycarbonate membrane inserts in a 24-well plate. Scale bar=20 μm (n=4, *p < 0.05).

C. The invasion ability of HeLa miR-552 mimic and its control cells was performed utilizing polycarbonate membrane inserts in a 24-well plate. Scale bar=20 μm (n=4, *p < 0.05).

D. The invasion ability of SiHa miR-552 mimic and its control cells was performed utilizing polycarbonate membrane inserts in a 24-well plate. Scale bar=20 μm (n=4, *p < 0.05).

Supplementary Tables

Supplementary Table 1. Clinicopathological features of 30 cervical cancer patients

Characteristics		Total n=30
Age(year)	≤45	9
	>45	21
Tumor size(cm)	<4	14
	≥4	16
Stromal metastasis	Yes	16
	No	14
Lymph node metastasis	Yes	11
	No	19
